# Development and Preliminary Evaluation of an Internet-Based Healthy Eating Program: Randomized Controlled Trial

**DOI:** 10.2196/jmir.3534

**Published:** 2014-10-10

**Authors:** Katy Tapper, Gabriela Jiga-Boy, Gregory R Maio, Geoffrey Haddock, Michael Lewis

**Affiliations:** ^1^Department of PsychologyCity University LondonLondonUnited Kingdom; ^2^Department of PsychologySwansea UniversitySwanseaUnited Kingdom; ^3^School of PsychologyCardiff UniversityCardiffUnited Kingdom; ^4^College of EngineeringSwansea UniversitySwanseaUnited Kingdom

**Keywords:** social values, diet, fruit, vegetables, saturated fat, added sugar, motivation, Internet, health promotion, psychology

## Abstract

**Background:**

The HealthValues Healthy Eating Programme is a standalone Internet-based intervention that employs a novel strategy for promoting behavior change (analyzing one’s reasons for endorsing health values) alongside other psychological principles that have been shown to influence behavior. The program consists of phases targeting motivation (dietary feedback and advice, analyzing reasons for health values, thinking about health-related desires, and concerns), volition (implementation intentions with mental contrasting), and maintenance (reviewing tasks, weekly tips).

**Objective:**

The aim was to examine the effects of the program on consumption of fruit and vegetables, saturated fat, and added sugar over a 6-month period.

**Methods:**

A total of 82 females and 18 males were recruited using both online and print advertisements in the local community. They were allocated to an intervention or control group using a stratified block randomization protocol. The program was designed such that participants logged onto a website every week for 24 weeks and completed health-related measures. Those allocated to the intervention group also completed the intervention tasks at these sessions. Additionally, all participants attended laboratory sessions at baseline, 3 months, and 6 months. During these sessions, participants completed a food frequency questionnaire (FFQ, the Block Fat/Sugar/Fruit/Vegetable Screener, adapted for the UK), and researchers (blind to group allocation) measured their body mass index (BMI), waist-to-hip ratio (WHR), and heart rate variability (HRV).

**Results:**

Data were analyzed using a series of ANOVA models. Per protocol analysis (n=92) showed a significant interaction for fruit and vegetable consumption (*P*=.048); the intervention group increased their intake between baseline and 6 months (3.7 to 4.1 cups) relative to the control group (3.6 to 3.4 cups). Results also showed overall reductions in saturated fat intake (20.2 to 15.6 g, *P*<.001) and added sugar intake (44.6 to 33.9 g, *P*<.001) during this period, but there were no interactions with group. Similarly, there were overall reductions in BMI (27.7 to 27.3 kg/m^2^, *P*=.001) and WHR (0.82 to 0.81, *P*=.009), but no interactions with group. The intervention did not affect alcohol consumption, physical activity, smoking, or HRV. Data collected during the online sessions suggested that the changes in fruit and vegetable consumption were driven by the motivational and maintenance phases of the program.

**Conclusions:**

Results suggest that the program helped individuals to increase their consumption of fruit and vegetables and to sustain this over a 6-month period. The observed reduction in fat and sugar intake suggests that monitoring behaviors over time is effective, although further research is needed to confirm this conclusion. The Web-based nature of the program makes it a potentially cost-effective way of promoting healthy eating.

## Introduction

 A diet that is high in saturated fat and added sugars and low in fruit and vegetables is associated with a range of chronic diseases, including cardiovascular disease, cancer, and diabetes [1-5]. However, such a diet is typical for a large proportion of European and North American adults [3,6-8], and lifestyle-related diseases are now the leading cause of death globally [9]. Therefore, dietary improvement has become a priority for many Western governments [10].

One way of promoting a more healthy diet is via Internet-based intervention. This has a range of potential advantages [11], including the ability to incorporate interactive and tailored features into a program that is fully automated. This makes it a potentially cost-effective approach. Indeed, a number of fully automated Internet interventions have shown positive effects on diet. For example, compared with control groups, 4 studies have found significant reductions in fat intake up to 8 months from baseline [12-15], 3 studies have found significant increases in fruit and vegetable consumption up to 15 months [15-17], and 1 study has found a significant reduction in added sugar intake at 4 months, although not at 8 months [15].

Although these results offer a useful first step in understanding the efficacy of Internet-based health promotion interventions, most of them draw on the same set of behavior change theories to guide content development. In particular, social cognitive theory, the theory of reasoned action / planned behavior, and the transtheoretical model are frequently used [18]. Theory is a powerful tool for effective interventions [18], but these models sometimes lack empirical support and specific details about how to actually change behavior [19-21]. Additionally, they do not always encompass the latest research findings.

This paper describes the initial evaluation of a new, fully automated Internet-based healthy eating intervention: the HealthValues Healthy Eating Programme. This program differs from previous Web interventions in its use of novel behavior change techniques. In developing the HealthValues Programme, we used a more bottom-up approach, employing a selection of distinct, brief interventions that have been shown to influence behavior. There are a wide range of such techniques in the research literature, but these often fail to be translated into practice. As such, the strategies we selected can be viewed as a starting point rather than a comprehensive selection.

The first strategy involved asking individuals to spend 5 minutes thinking about why the value of health is important or unimportant to them. There is evidence that social values (eg, equality, helpfulness) often lack cognitive support. In other words, although individuals believe them to be important, they have not necessarily thought about why they are important [22]. This means that they tend to behave in accordance with the value only when it is relatively easy to do so. However, asking individuals to think about the reasons underpinning social values can help them build cognitive support for these values and, in turn, promote more value-consistent behavior [23]. Recent research has suggested that health values also lack cognitive support, to the extent that thinking about reasons for health can have a positive influence on eating behaviors [24]. Given that this lack of cognitive support was evident across a range of social groupings and regardless of whether individuals lead healthy or unhealthy lifestyles, it suggests that this very simple strategy may be beneficial for a large number of individuals.

The second and third strategies asked individuals to spend 5 minutes considering (1) their desires and aspirations in relation to their health together with how achieving these would make them feel and (2) their concerns in relation to their health alongside how failing to avoid these would make them feel. These strategies map onto techniques commonly employed in motivational interviewing (MI) [25]. MI aligns with the principles of self-determination theory (SDT) [26] and has been shown to be effective in promoting dietary change [27]. These 2 strategies also draw on suggestions that affective messages may result in greater behavioral change than cognitive-based messages [28,29], but consistent with MI and SDT, these strategies take a nondirective approach.

The fourth strategy consists of implementation intentions with mental contrasting. Implementation intentions are specific plans of when, where, and how someone will change their behavior. They are believed to work by (1) increasing the accessibility of the situational cue that is relevant to the target behavior and (2) increasing the efficiency with which one performs the target behavior in the presence of the situational cue [30]. There is considerable evidence that implementation intentions can help promote behavior change [31,32]. In the present study, implementation intentions were employed in combination with mental contrasting. Mental contrasting involves thinking about both positive outcomes following successful behavior change as well as obstacles that might stand in the way of behavior change [33]. Mental contrasting with implementation intentions has been shown to reduce unhealthy snacking to a greater degree than either strategy in isolation [33] and has also been shown to increase fruit and vegetable consumption over a 2-year period [34].

To enhance the efficacy of the implementation intentions, we also utilized evidence about moderators by including a number of other features. These were the use of an “if...then...” format [35], use of self-formulated rather than assigned implementation intentions [36], visualization of the implementation intention [33], the formation of just 1 implementation intention at a time [37,38], emailed reminders of the implementation intention [18], the opportunity to review and modify the implementation intention in subsequent weeks [39,34], and a limited amount of tailored feedback aimed at promoting self-efficacy and autonomy [40].

The fifth strategy was the use of tailored dietary feedback in conjunction with standard health promotion advice [41,42]. Participants were provided with estimates of their intake of saturated fat, added sugar, and fruit and vegetables, along with government intake recommendations, information on the health consequences of high or low intake, and some simple strategies for adjusting one’s diet. Although this component of the intervention was similar to what might be contained in an intervention with a more educational approach, an awareness of one’s own diet and how it might be improved was deemed to be a prerequisite for subsequent change [43].

Finally, the program also incorporated weekly tips during the last phase. These were primarily aimed at maintaining user engagement [44] rather than promoting behavior change per se. They were designed to be light-hearted and engaging, but were also evidence-based.

Drawing on the model of action phases [45], these strategies were divided into a motivational phase (dietary feedback, reasons for health values, health-related desires and aspirations, health-related concerns) and a volitional phase (implementation intentions). This was followed by a maintenance phase during which participants could repeat or review previous tasks and information and could also access the Tip of the Week. We evaluated the program over a 6-month period through the use of laboratory-based measures taken at baseline, 3 months, and 6 months, and via weekly online measures. The intervention group was compared with a control group who completed the laboratory and online measures, but not the intervention strategies. The main aim of the study was to examine the effects of the program on different types of health-related eating behaviors, those that require engagement (eating more fruit and vegetables) and those that require disengagement (eating less saturated fat and added sugar). However, we were also interested in examining the spillover effects to other health-related behaviors (eg, physical activity, alcohol consumption, smoking) [46].

## Methods

### Sample Size

Given that this study served as an initial test of the program, there were no comparable studies on which to base sample size calculations. That said, our sample size was informed by our previous research that examined the effects of 1 of the intervention components (thinking about reasons for values) on eating behavior over a 7-day period [24]. The eating behavior measure showed a mean difference between groups of 0.92 and a standard deviation of 1.51, meaning that at 80% power, 44 participants per group would be needed to detect a significant difference (2-tailed, *P*<.05). Assuming an attrition rate of no more than 15% [47], we concluded that a sample size of 100 would be appropriate for this trial.

### Participants

Participants were recruited using both online and print advertisements in the local community. These included posters and flyers in local shops and community facilities, and advertisements on social media sites, email networks, and in local newspapers. The advertisements stated that the study team were looking for individuals to test a new online healthy eating program and noted that individuals would be reimbursed for participation. The study’s website address (which included a full participant information sheet) was included in the advertisement. See Multimedia Appendices 1 and 2 for study home page and information sheet.

**Figure 1:**
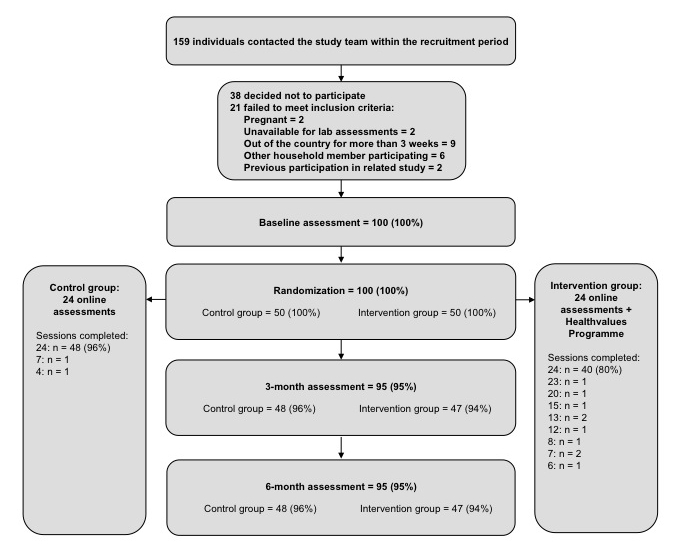
Flow of participants through the study.

As inclusion criteria, we stipulated that participants be aged 18 or older and able to comply with the study procedures (ie, attend the laboratory appointments and complete the weekly online sessions). Other exclusion criteria were pregnancy, being out of the country for more than 3 weeks during the study period, another household member already participating, and participation in a previous related study. A total of 159 individuals contacted the study team during the recruitment period. Of these, 38 decided not to take part or failed to respond to subsequent communications and 21 did not meet inclusion criteria. Figure 1 shows the flow of participants through the study. Of the 100 participants recruited, 82 were females and 18 were males. Mean age was 39 (SD 14) years and mean body mass index (BMI) was 27.68 (SD 5.73) kg/m^2^. A total of 23 participants were dieting to lose weight. Participants were predominantly white (93.0%, 93/100) and most had English or Welsh as a first language (94.0%, 94/100) and were well-educated (63.0%, 63/100 to degree level).

### Study Design and Procedure

The study received ethics approval from Swansea University Psychology Department Ethics Committee. Informed consent was collected by researchers at the first laboratory assessment (described subsequently). Although the study was a randomized controlled trial design, given its exploratory nature, the trial was not registered.

Laboratory measures were taken at baseline (February to April 2012), and at 3 months (May to July 2012) and 6 months (August to October 2012) postbaseline by GJB and a second research assistant, both of whom were blind to group allocation. Following baseline assessment, GJB emailed KT details of each participant’s dieting status and fruit and vegetable consumption. KT then allocated participants to an intervention or control (“monitoring”) group using a stratified block randomization protocol on the basis of dieting status (dieting versus nondieting) and fruit and vegetable consumption (≥5 a day versus <5 a day). Block size was 2 and random numbers were generated in Excel. KT then emailed the participant details of their user ID and password and they were informed of their group allocation the first time they logged on. Although participants were not blind to group allocation, they were informed that both the “experimental” group and the “monitoring” group would monitor eating behaviors and that this had been shown to be useful for reaching health goals. Participants in the control group were offered the opportunity to complete the program tasks at the end of the study.

All participants were asked by automated email to log onto the study website every week on 24 separate occasions to complete measures (intervention and control group) and program tasks (intervention group only). Each session could be accessed 6 days after completion of the previous session. Once the session became available, the participant was sent an email asking them to log in to complete it. Up to 3 automated reminders were emailed 2, 4, and 6 days later to participants who failed to complete the session. After completion of each session, the participant was sent an automated email thanking them and reminding them to log in again the following week. If participants failed to log in for 3 weeks, GJB attempted to contact them by phone and then email to establish whether they still wanted to participate in the online sessions and, if not, to assure them that we would still be keen for them to attend the laboratory assessments.

Each participant received £10 (approximately US $17) for attending the first laboratory session, £25 (US $42) for the second, and £50 (US $84) for the third. Additionally they received £2 (US $3) per session for completing the first 10 online sessions, £2.50 (US $4) per session for completing the next 10 online sessions, and £5 (US $8) per session for completing the last 4 online sessions. Thus, participants could receive up to £150 (US $253) for completing all laboratory and online sessions. Money for completing the online sessions was given at the final laboratory assessment and amounts allocated were indicated in emails sent to prompt, remind, and thank participants. In a further effort to limit attrition, participants received small gifts (a fabric bag and a mouse pad) at the first and second laboratory assessments. These were branded with the HealthValues logo.

### Measures

#### Outcome Measures

Primary outcome measures were intake of (1) saturated fat, (2) added sugar, and (3) fruit and vegetables. These were assessed in a laboratory using the Block Fat/Sugar/Fruit/Vegetable screener, a 55-item food frequency questionnaire (FFQ) adapted from a longer version that has been shown to have good reliability and validity [48,49]. The FFQ included questions about both frequency and quantity of intake. It was developed in North America and for our purposes adapted for use in the UK. Because the questionnaire often referred to quantities in terms of “cups,” participants were also given 4 UK measuring cups (1 cup, 1/2 cup, 1/4 cup, 1/8 cup) to assist them with their portion estimates when completing the questionnaire.

Secondary outcome measures were BMI, waist-to-hip ratio (WHR), heart rate variability (HRV), smoking status, smoking frequency, quantity of alcohol consumed, binge drinking, physical activity, dietary behaviors, and additional online assessments of saturated fat, added sugar, and fruit and vegetable intake. BMI, WHR, and HRV were assessed in the laboratory by trained researchers. These physiological measures provide an objective assessment of health status [50]. For example, HRV is a surrogate measure of cardiac control via the autonomic nervous system and can be considered to be a measure of cardiac fitness. Less favorable HRV profiles are associated with hypertension, cardiovascular disease, and aging [51], whereas physical activity has a positive effect on HRV profile [52,53]. In this study, we quantified HRV using the common statistical indexes standard deviation of cardiac (“RR”) interval (SDRR) and root mean square of successive differences (RMSSD), which reflect overall HRV and short-term (respiratory-mediated) HRV, respectively [54]. Higher scores represent better cardiac control.

Alcohol consumption was measured in the laboratory using a questionnaire designed to capture episodes of binge drinking as well as typical drinking behaviors [55]. It contained 4 items asking about frequency of consumption and number of units consumed for both usual consumption and for days when the respondent consumed larger-than-usual quantities. The questionnaire was scored by converting frequencies to drinks per week and then multiplying frequency by number of units to obtain the number of units consumed per week from usual drinking. To compute additional units consumed from larger-than-usual episodes, the usual number of units consumed was first subtracted from the larger-than-usual number of units. This gave the number of additional units consumed on these occasions. This number was then multiplied by the larger-than-usual frequency to obtain a figure for the additional number of units consumed per week from more-than-usual drinking. The 2 figures were then added together to obtain the overall number of units consumed per week. In-line with British government recommendations, binge drinking was defined as 8 or more units per day for men and 6 or more units per day for women [56]. Where quantities consumed for either usual consumption or larger-than-usual consumption met these criteria, they were coded as an episode of binge drinking.

Smoking was assessed in the laboratory by asking participants whether they smoked cigarettes and, if yes, the number they usually smoked either per day, per week, or per month. Scores were recorded into number smoked per week.

Physical activity was assessed online at sessions 1, 8, 12, and 24 using the short version of the International Physical Activity Questionnaire (IPAQ) [57]. Participants indicated on how many days and for how long they had engaged in vigorous activity, moderate activity, and walking during the previous week. These scores were converted into total number of metabolic equivalent of task (MET) units expended per day [58].

In addition to the laboratory assessments, saturated fat, added sugar, and fruit and vegetable consumption were also assessed online at sessions 1, 8, 12, and 24 using a validated UK FFQ [59]. Respondents recorded the frequency with which they consumed 63 common food items over the previous month. The FFQ has been shown to have good test-retest reliability [60], as well as good convergent validity with 10-day weighed records [61] and with 24-hour dietary records [59]. The FFQ has also been shown to possess good construct validity [62].

To compute daily intake of saturated fat and added sugar, the proportions of these macronutrients in each of the 63 foods were calculated based on data provided by the British Food Standards Agency [63,64]. Each participant’s daily intake of each food was then computed by multiplying frequency of consumption by average portion size. Average portion sizes were based on Bingham and Day [65] and the British Food Standards Agency [64]. Finally, the quantities of saturated fat and added sugar consumed were calculated by multiplying daily intake values of the various food types by the proportion of saturated fat/added sugar in each food. These were then summed across the 63 foods to provide daily total consumption of saturated fat and added sugar for each participant.

Two additional questions were used in the calculation of fruit and vegetable consumption. These were the number of portions of fruit (excluding fruit juice), and the number of portions of vegetables (excluding potatoes, beans, and lentils) eaten on a typical day during the previous week. Examples of portions were provided. These scores were combined with scores from items relating to fruit juice and beans/lentils from the FFQ to compute daily servings of fruit and vegetables. In-line with UK guidelines, juice and beans/lentils were counted as a maximum of 1 serving a day each.

Dietary behaviors were assessed at the start of each of the 24 online sessions using a questionnaire that was developed for the project. This consisted of 17 items associated with standard dietary advice related to consumption of saturated fat, added sugar, and fruit and vegetables (eg, reducing the number of teaspoons of sugar added to hot drinks, cereals, and desserts; replacing red meat with white meat or fish). The items were a mix of quantitative (eg, number of high fat snacks during the previous week) and categorical (eg, type of milk usually consumed). To reduce respondent burden, after the first session participants were presented with their responses from the previous session and asked to simply adjust their answers where they had made a dietary change. The questionnaire was scored by calculating the number of positive versus negative changes made since the previous session (–17 to +17).

All online questionnaires were tested for usability before the study. Questionnaires and items were presented in the same order for each participant and participants needed to complete all items before progressing to the next screen. Adaptive questioning was used for the IPAQ.

#### Demographic Measures

Details of participants’ gender, age, level of education, and first language were collected at the first online session.

#### Additional Measures

Data relating to potential mediators (habits, intentions, self-efficacy, anticipated emotions), moderators (need for affect, need for cognition, behavioral approach system sensitivity, behavioral inhibition system sensitivity, environmental change), and process measures (poststudy feedback questionnaires and telephone interviews) were also collected, but these are not discussed in the present paper.

### Intervention

The intervention was tested for usability before the study. At all sessions, intervention components were delivered after assessment measures. The intervention components are detailed in Multimedia Appendix 3. For information purposes, Multimedia Appendix 1 also shows how the components relate to Michie and colleagues’ recommended taxonomy of behavior change techniques [66]. Further details of the intervention components can be obtained from the first author.

### Statistical Analysis

Baseline characteristics of the 2 groups were compared using *t* tests and chi-square tests. Given the exploratory nature of the trial, intention-to-treat analyses were conducted on primary outcomes only. Missing data were replaced by calculating the mean change from previous observations in the control group and adding or subtracting this figure from the previous observation relating to the missing data point. To examine changes in time over the 6-month period, ANOVA models with time as an independent variable were employed for the main analyses. Thus, a series of 3×2 mixed ANOVA models were used to examine the effects of the intervention on laboratory-measured intake of (1) saturated fat, (2) added sugar, and (3) fruit and vegetables. Independent variables were time (baseline, 3 months, 6 months) and group (control, intervention). There were 7 outliers (defined as greater than 3.5 SDs from the mean) and the analysis was conducted both with these unchanged and by adjusting them to 3.5 SDs from the mean.

Per protocol analysis was conducted on all primary and secondary outcomes by including only those participants who completed all 3 laboratory assessments as well as 12 or more of the 24 online sessions (for laboratory measures) or all 24 online sessions (for online measures). Although the samples for such analyses are subject to bias, they are an important means of examining intervention efficacy in exploratory trials. A series of 3 (time) × 2 (group) mixed ANOVA models were used to examine effects on laboratory-based measures whereas 4 (time) × 2 (group) ANOVA models were used for online measures. Analyses were conducted with outliers (defined as 3.5 SDs from the mean) both included and excluded. Fisher exact test was used to examine smoking status and chi-square test was used for binge drinking status.

To examine the effects of the individual intervention strategies employed in the motivational phase, change scores were calculated using the dietary behaviors questionnaire. These were computed using figures from the session in which the strategy was employed and 2 sessions later (eg, change between sessions 1 and 3, see Multimedia Appendix 1 for details of strategies). Change score was then employed as the dependent variable in a 2 (condition) × 4 (strategy) mixed ANOVA.

## Results

### Baseline Characteristics

Analysis of baseline characteristics showed that the intervention and control groups were well matched across a range of variables (see Table 1).

**Table 1:** Baseline characteristics of the intervention and control groups (N=100).

Variable	Control group (n=50)	Intervention group (n=50)	*P* value
Gender (female), n (%)	42 (84)	41 (82)	.79^b^
Age (years), mean (SD)	37.7 (13.2)	41.1 (14.1)	.21^c^
BMI (kg/m^2^), mean (SD)	28.1 (5.8)	27.1 (5.7)	.40^c^
Dieting status (dieting), n (%)	11 (22)	12 (24)	.81^b^
Education level (degree level or higher),^a^ n (%)	29 (58)	34 (68)	.86^b^
First language (English/Welsh), n (%)	49 (98)	45 (90)	.09^b^
Ethnic background (white British), n (%)	42 (84)	34 (68)	.32^b^

^a^ Highest level of educational attainment coded as GCSEs, A-levels, degree (or equivalent), still studying or other.

^b^ *t* test.

^c^ Chi-square test.

### Intention-to-Treat Analyses

Descriptive and inferential statistics for intention-to-treat analyses (without outlier adjustment) are shown in Table 2. The results suggest that although both groups showed significant reductions in saturated fat and added sugar over the 6-month period, participants allocated to the intervention group did not show greater improvements than those allocated to the control group. There was no overall change in fruit and vegetable consumption over time, but a trend toward an increase in the intervention group relative to the control group (small to medium effect size). Repeating the analyses with outlier adjustment showed near identical results.

**Table 2:** Means (SDs) and results from ANOVA models for intake of saturated fat, added sugar, and fruit and vegetables at baseline, 3 months, and 6 months in the intervention and control groups, for the intention-to-treat analysis.

Variable and time		Group, mean (SD)		Effects for time			Effects for time × group		
		Control (n=50)	Intervention (n=50)	*F*_1, 98_	*P*	Partial η^2^	*F*_1, 98_	*P*	Partial η^2^
**Saturated fat (grams)**				35.9	<.001	0.27	0.8	.36	0.01
	Baseline	21.4 (8.9)	19.7 (9.6)						
	3 months	17.3 (8.3)	16.1 (7.7)						
	6 months	15.9 (6.6)	15.7 (9.9)						
**Added sugar (grams)**				8.6	.004	0.08	0.2	.62	0.00
	Baseline	47.6 (34.0)	43.2 (42.0)						
	3 months	36.7 (30.4)	30.3 (25.5)						
	6 months	38.5 (37.6)	30.5 (37.0)						
**Fruit and vegetables (cups)**				0.0	.98	0.00	3.1	.08	0.03
	Baseline	3.6 (1.5)	3.7 (1.7)						
	3 months	3.5 (1.9)	3.8 (1.7)						
	6 months	3.3 (1.5)	3.9 (1.6)						

### Per Protocol Analyses

Descriptive and inferential statistics for continuous primary and secondary outcome measures collected at laboratory sessions are shown in Table 3. Over the 6-month period, participants in both groups showed comparable declines in saturated fat intake, added sugar intake, BMI, and WHR. For fruit and vegetable intake, the intervention group showed significant increases relative to the control group. Follow-up independent *t* tests indicated no difference in fruit and vegetable consumption between the intervention and control groups at baseline and 3 months (*t*_90_=0.31, *P*=.78 and *t*_90_=1.01, *P*=.28, respectively), but significantly greater intake in the intervention group at 6 months (*t*_90_=2.30, *P*=.02). For the RMSSD HRV measure there was a trend toward a significant group × time interaction, but no main effect of time. SDRR HRV and total alcohol intake did not change over time and were not influenced by group status. The same pattern of results occurred when these analyses were repeated but with outliers excluded.

**Table 3:** Means (SDs) and results from ANOVA models for laboratory-assessed primary and secondary outcomes at baseline, 3 months, and 6 months in the intervention and control groups, for the per protocol analyses.

Variable and time		Group, mean (SD)		Effects for time			Effects for time × group		
		Control (n=47)^a^	Intervention (n=45)^b^	*F*_1, 90_^c^	*P*	Partial η^2^	*F*_1, 90_^c^	*P*	Partial η^2^
**Saturated fat (grams)**				28.7	<.001	0.24	1.2	.23	0.01
	Baseline	21.0 (8.9)	19.3 (8.9)						
	3 months	16.7 (8.0)	16.2 (7.3)						
	6 months	15.5 (6.4)	15.7 (9.6)						
**Added sugar (grams)**				7.2	.009	0.07	0.1	.76	0.00
	Baseline	46.7 (34.3)	42.3 (43.0)						
	3 months	35.8 (30.5)	30.4 (26.1)						
	6 months	37.2 (38.1)	30.4 (38.5)						
**Fruit and vegetables (cups)**				0.3	.57	0.00	4.0	.048	0.04
	Baseline	3.6 (1.4)	3.7 (1.7)						
	3 months	3.4 (1.7)	3.8 (1.7)						
	6 months	3.4 (1.5)	4.1 (1.6)						
**Alcohol (units per week)**				1.6	.20	0.02	0.2	.69	0.00
	Baseline	6.4 (5.6)	6.3 (6.2)						
	3 months	6.8 (7.2)	6.7 (6.9)						
	6 months	7.2 (7.5)	6.7 (7.3)						
**BMI (kg/m** ^**2**^ **)**				11.2	.001	0.11	0.1	.93	0.00
	Baseline	28.4 (5.8)	27.0 (5.9)						
	3 months	28.3 (5.9)	26.8 (5.7)						
	6 months	28.0 (5.9)	26.6 (5.9)						
**WHR**				7.2	.009	0.07	0.0	.71	0.00
	Baseline	0.82 (0.09)	0.82 (0.09)						
	3 months	0.81 (0.09)	0.82 (0.09)						
	6 months	0.81 (0.08)	0.81 (0.08)						
**HRV: SDRR (ms)**				1.4	.25	0.02	2.0	.13	0.02
	Baseline	45.0 (20.1)	49.6 (19.7)						
	3 months	46.4 (20.1)	47.8 (18.7)						
	6 months	46.1 (17.9)	43.1 (15.2)						
**HRV: RMSSD (ms)**				1.4	.24	0.02	2.9	.06	0.03
	Baseline	28.9 (14.6)	33.1 (19.6)						
	3 months	19.3 (15.3)	30.5 (16.3)						
	6 months	30.2 (15.4)	25.8 (12.9)						

^a^ For alcohol consumption, n=46 due to questionnaire completion error.

^b^ For alcohol consumption, n=44 due to questionnaire completion error.

^c^ For alcohol consumption, *F*_1, 88_.

For smoking status, there were 91 participants who provided data on smoking at all 3 laboratory assessments and completed at least 12 of the online sessions. At each of the 3 time points there was no difference in the proportion of smokers in the experimental group compared to the control group at baseline (control: n=6, experimental: n=2, *P*=.27), 3 months (control: n=4, experimental: n=1, *P*=.36), and at 6 months (control: n=4, experimental: n=3, *P*>.99. Smoking frequency was not analyzed due to the small number of smokers in the sample.

Analysis of binge drinking included 90 participants who provided data on alcohol consumption at all 3 laboratory assessments and completed at least 12 of the online sessions. Again, at each of the 3 time points, there was no difference in the proportion of individuals who engaged in binge drinking in the experimental group compared to the control group at baseline (control: n=25, experimental: n=23; χ^2^_1_=0.0, *P*=.84), 3 months (control: n=23, experimental: n=17; χ^2^_1_=1.1, *P*=.28) and 6 months (control: n=20, experimental: n=17; χ^2^_1_=0.2, *P*=.64).

Descriptive and inferential statistics for secondary outcome measures collected during the online sessions are shown in Table 4. Consistent with laboratory assessments, these show there were significant reductions in intake of saturated fat and added sugar over time, but that the extent of these reductions did not differ between intervention and control groups. Also consistent with laboratory assessments, the results show an increase in fruit and vegetable consumption among the intervention group relative to the control group. This was coupled with an overall increase in fruit and vegetable consumption over time. Follow-up independent *t* tests indicated no difference in fruit and vegetable consumption between the intervention and control groups at sessions 1, 8, and 12 (*t*_86_=0.19, *P*=.85; *t*_86_=1.64, *P*=.11; *t*_86_=1.48, *P*=.14, respectively), but significantly greater intake in the intervention group at session 24 (*t*_86_=2.45, *P*=.02). Additionally, the results showed no significant change in physical activity over time and no effect of the intervention on physical activity. The same pattern of results occurred when these analyses were repeated with outliers excluded.

**Table 4:** Means (SDs) and results from ANOVA models for secondary outcomes assessed online at sessions 1, 8, 12, and 24 in the intervention and control groups, for the per protocol analyses.

Variable and session		Group, mean (SD)		Effects for time			Effects for time × group		
		Control (n=48)^a^	Intervention (n=40)^b^	*F*_1, 86_^c^	*P*	Partial η^2^	*F*_1, 86_^c^	*P*	Partial η^2^
**Saturated fat (grams)**				7.8	.006	0.08	0.6	.43	0.01
	1	24.4 (9.9)	26.0 (15.4)						
	8	22.3 (10.6)	21.4 (13.0)						
	12	21.2 (10.4)	21.7 (11.4)						
	24	22.4 (10.0)	21.5 (9.1)						
**Added sugar (grams)**				8.41	.005	0.10	2.0	.16	0.02
	1	47.8 (43.6)	57.32 (74.5)						
	8	34.4 (22.7)	34.4 (32.3)						
	12	31.7 (21.4)	32.1 (25.3)						
	24	39.8 (27.0)	31.8 (19.4)						
**Fruit and vegetables (portions)**				5.6	.02	0.06	5.5	.02	0.06
	1	4.9 (2.1)	5.0 (2.0)						
	8	5.2 (2.4)	6.0 (2.3)						
	12	5.3 (2.8)	6.1 (2.2)						
	24	4.9 (2.3)	6.2 (2.7)						
**Physical activity (METS per week)**				0.2	.67	0.00	0.2	.69	0.00
	1	2857 (2320)	2432 (1626)						
	8	2534 (2290)	2138 (1522)						
	12	2932 (4270)	2420 (1966)						
	24	2985 (3525)	2350 (2344)						

^a^ For physical activity n=39 due to participants coding “don’t know.”

^b^ For physical activity n=37 due to participants coding “don’t know.”

^C^ For physical activity, *F*_1, 74_.

**Figure 2:**
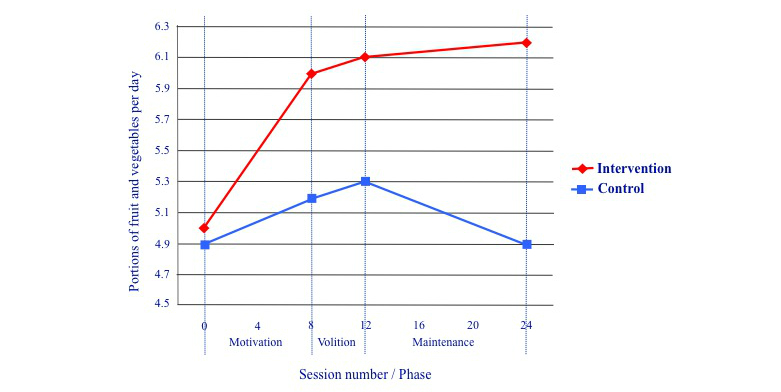
Portions of fruit and vegetables consumed in the intervention and control groups at the start and end of each program phase.

Figure 2 shows levels of fruit and vegetable consumption in the intervention and control groups at the start and end of each of the 3 program phases. As noted previously, follow-up analyses indicated that significant differences between intervention and control groups occurred at the fourth measurement point only (ie, session 24, the end of the third phase, *t*_86_=2.45, *P*=.02). These results, together with Figure 2, suggest that the most likely explanation for this effect is that it was driven primarily by the combination of motivation and maintenance phases. However, it is also possible that the maintenance phase played no part in the changes, but that the differences at session 24 were a result of the motivational phase continuing to exert effects over the 6-month period. Additionally, the data suggest that (in its position within the intervention) the volitional phase had no immediate impact (although a delayed impact cannot be ruled out). The pattern of results from the per protocol analysis were unchanged after repeating the analysis with only the intervention participants who had formed at least 1 volitional phase implementation intention related to the relevant outcome measure (fruit and vegetables: n=24; saturated fat: n=30; added sugar: n=32).

### Effects of Individual Strategies Employed in the Motivational Phase

**Figure 3:**
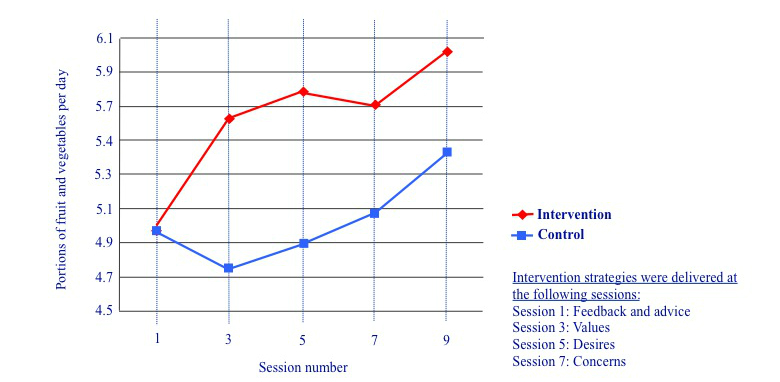
Portions of fruit and vegetables consumed in the intervention and control groups during the motivational phase.

For analysis of motivational phase strategies, all participants who completed the first 9 online sessions were included (control: n=47; intervention: n=46). Because fruit and vegetable consumption was improved by the intervention, we conducted exploratory analyses examining changes in fruit and vegetable consumption in the intervention and control groups in the 2-week period following the delivery of each of the 4 different program components (see Figure 3). There was no main effect of strategy (*F*_1, 91_=0.53, *P*=.47, partial eta-squared=0.01) or condition (*F*_1, 91_=0.87, *P*=.47, partial eta-squared=0.01) and no significant interaction between strategy and condition (*F*_1, 91_=2.88, *P*=.09, partial eta-squared=0.03), although the latter results are marginal. These results suggest that the increases in fruit and vegetable consumption seen in the intervention group were brought about by a combination of intervention components in both the motivational and maintenance phases. Figure 3 suggests that the strategy employed in session 1 (tailored feedback and advice) may have been particularly useful in eliciting change, although further research is needed to confirm this.

## Discussion

Results of the per protocol analysis indicated that the HealthValues Healthy Eating Programme brought about significant increases in fruit and vegetable consumption relative to a control group. These equated to approximately 0.75 cups, or 1.3 portions of the recommended 5 or more portions per day. The results also suggested that these increases were brought about primarily by strategies employed in the motivational and maintenance phases of the program, rather than the implementation intentions employed in the volitional phase. Thus, it may be that low fruit and vegetable consumption among this particular group was limited primarily by motivation rather than any difficulties in implementing the behavior; when we increased motivation, it had a direct effect on consumption.

In contrast, although the program was associated with a decrease in saturated fat and added sugar consumption, these effects were comparable to those found in the control condition. Unlike increasing fruit and vegetable intake, which involves introducing additional foods into the diet, reducing fat and sugar entails cutting back. As such, intake may be influenced by additional factors that may not be as amenable to motivational strategies. In particular, consumption of high fat and sugar foods may be habitual and carried out with a degree of automaticity [67,68]. Because habits tend to be resistant to changes in attitude [69], motivational strategies alone may be ineffective in eliciting a reduction in these forms of consumptive behavior. Additionally, foods that are high in fat and sugar may be the target of cravings [70]. Again, motivational strategies may not be sufficient to overcome such cravings. Thus, techniques specifically designed to target habits and cravings might usefully be incorporated into future versions of the program.

The results did, however, show overall reductions in intake of saturated fat and added sugar among both groups by approximately 4.7 and 11.4 grams per day, respectively. These findings are consistent with the physiological data that showed significant reductions in BMI and WHR. Given that our recruitment method targeted individuals who wanted to improve their diet, it is possible that these changes would have occurred even in the absence of study participation. However, this seems unlikely given the general trend for weight to increase over time [71] and the fact that these data were collected over an extended (6-month) period. Instead, we would suggest that these changes might have been brought about by the monitoring component of the study, particularly the weekly brief diet questionnaire that mapped directly onto dietary advice. This questionnaire may have increased participants’ knowledge of how to cut back on fat and sugar. It may also have increased attitude accessibility, the ease with which attitudes are retrieved from memory [72]. If intake of fat and sugar are determined by relatively weak habits, increased accessibility of negative attitudes toward fat and sugar may have been sufficient to disrupt automatic behaviors. Further research is needed to confirm this. It would also be important to control for the effects of researcher contact. In the current study, it is possible that the laboratory assessments, together with the incentives, may have inadvertently led to participants trying to please the researchers. These may have contributed in some small part to the overall reductions in fat and sugar intake.

The absence of effects for implementation intentions are at odds with previous non-Internet interventions [34,73], but are in-line with several other Internet-based studies [74-77]. One explanation is that participants had already formed action plans in response to the monitoring component of the study, making it difficult for the implementation intentions to bring about further change. This interpretation is consistent with other research showing implementation intentions to be less effective among individuals who are already good at action planning [78]. It also has implications for the development of interventions; because longer interventions may increase rates of drop out, it is important that all strategies employed make a unique contribution to behavior change. However, an alternative explanation is that the fruit and vegetable-related implementation intentions helped sustain behavior change [34]. A weakness of the current study is that it is unable to distinguish between these possibilities or to identify with precision the components that are responsible for the effects. In future work, it would be helpful to compare different versions of the program to help determine which components are important and which may be redundant.

The benefits of participation did not generalize to behaviors that were not directly targeted by the program; there were no significant spillover effects on levels of physical activity, alcohol consumption, smoking, or HRV, either between groups or over time. Although some research has suggested that health improvements may show spillover effects to other health-related behaviors [46], the results of this study suggest that effects are restricted to behaviors that are targeted.

In future research, it would be important to trial the program in the absence of incentives for session completion. Given the high rates of attrition in online interventions [79], we incorporated these incentives to enable a proper initial evaluation of the program. However, a trial without these incentives would help indicate natural attrition and allow for calculations of cost-effectiveness.

It is also important to examine the effects of the program with different populations. In the current study, we recruited participants who were interested in improving their diet. Thus, they were a group who were already reasonably motivated (as indicated by a baseline mean of 4.16 on a scale of 1 to 5 for intention to eat a healthy diet). It is possible that the motivational strategies would have been more effective among a less motivated group of individuals who might be accessed via workplace settings, for example.

In conclusion, the HealthValues Healthy Eating Programme significantly increased fruit and vegetable consumption among users. Future research comparing different versions of the program should help to identify more accurately the elements that were responsible for this effect. It seems likely that the monitoring component of the study also brought about reductions in intake of saturated fat and added sugar, although further research is needed to confirm this. Given that the program is fully automated, it represents a potentially cost-effective way of promoting healthy eating.

## Multimedia Appendix 1

HealthValues Healthy Eating Programme homepage.

## Multimedia Appendix 2

Study information sheet provided to participants.

## Multimedia Appendix 3

Intervention components used in the HealthValues Programme together with most closely aligned categories of behaviour change technique according to the Behavior Change Technique Taxonomy.[66] Shaded rows indicate optional components.

## Multimedia Appendix 4

CONSORT-EHEALTH checklist V1.6.2 [80].

## Abbreviations

BMI: body mass index
FFQ: food frequency questionnaire
HRV: heart rate variability
MET: metabolic equivalent of task
MI: motivational interviewing
RMSSD: root mean square of successive differences
SDRR: standard deviation of RR
SDT: self-determination theory
WHR: waist-to-hip ratio
