# Supplementary figures and images for: Development and Preliminary Evaluation of an Internet-Based Healthy Eating Program: Randomized Controlled Trial
Source: J Med Internet Res. 2014 Oct 10;16(10):e231. doi: 10.2196/jmir.3534 (PMC4210956; doi:10.2196/jmir.3534)

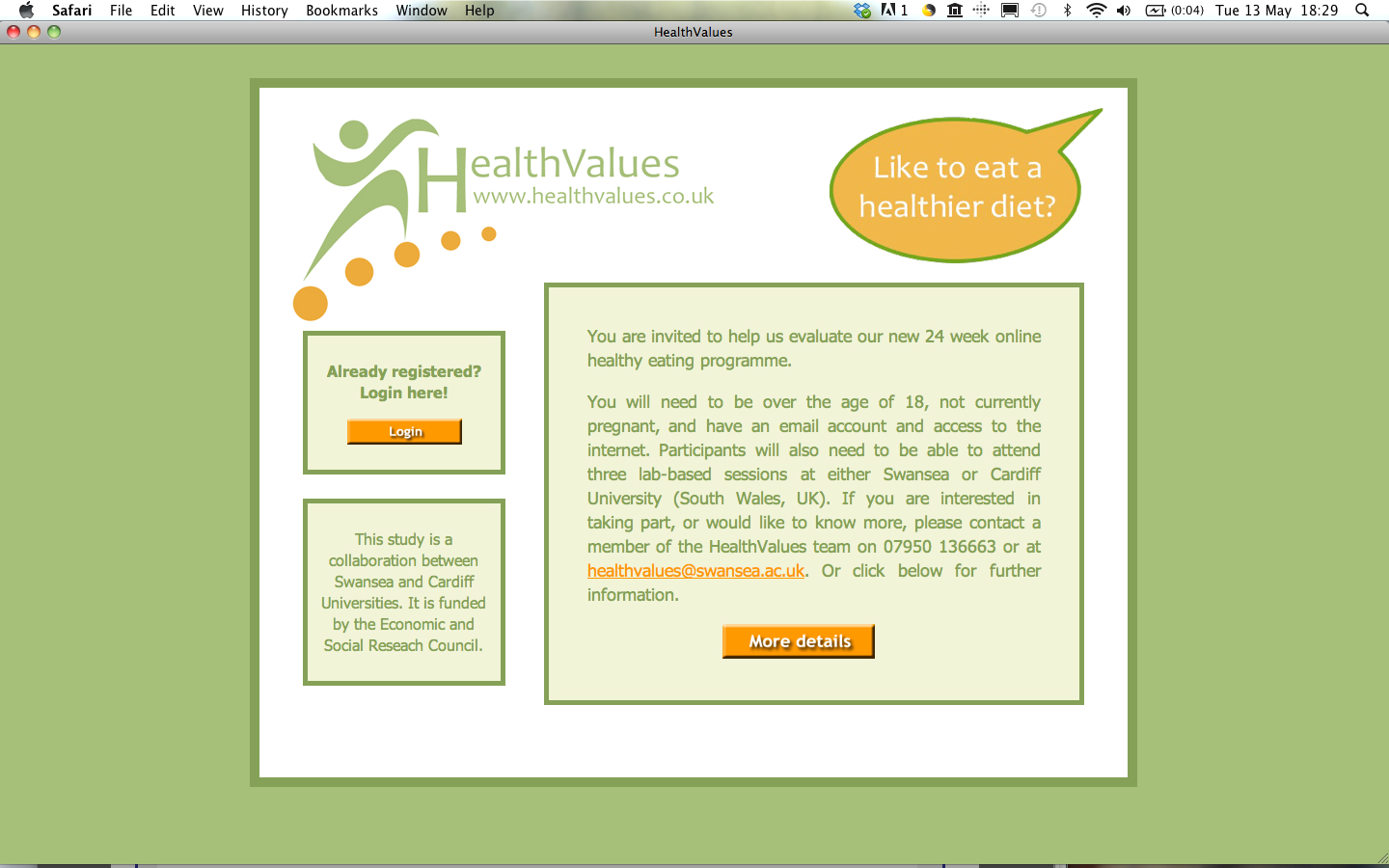

Supplement: Supplementary file 1 [file jmir_v16i10e231_app1.jpg]
