# Supplementary material for: Development and Preliminary Evaluation of an Internet-Based Healthy Eating Program: Randomized Controlled Trial
Source: J Med Internet Res. 2014 Oct 10;16(10):e231. doi: 10.2196/jmir.3534 (PMC4210956; doi:10.2196/jmir.3534)
Supplement: Supplementary file 2 [file jmir_v16i10e231_app2.pdf]

HealthValues: Evaluation of a Web-Based Healthy Eating Programme
Participant Information Sheet

You are being invited to take part in a research study. Before you decide whether or not to participate, it is important you understand what it involves. Please read the following information carefully.

What is the purpose of the research?
We are developing a web-based programme to help individuals lead healthier lifestyles. The research is being carried out to see whether the programme is effective and how it might be improved.

Who is carrying out the research?
The research is being led by Dr Katy Tapper, Professor Greg Maio, Professor Geoff Haddock and Dr Mike Lewis at Swansea and Cardiff Universities. The research assistants are Dr Gabriela Jiga-Boy and Ms Clare Clement. The research is funded by the Economic and Social Research Council and has received ethics approval from Swansea University Psychology Department Ethics Committee.

Can anyone take part?
You need to be over the age of 18, not currently pregnant and have an email account and access to the internet. If you have any conditions that affect your diet (e.g., diabetes, eating disorder) we also recommend you contact your GP before making any dietary changes.

What happens if I agree to take part?
You will be asked to attend a session at either Cardiff or Swansea University (whichever is more convenient for you) where a researcher will take a range of physiological measures. These will consist of weight, height, hip-to-waist ratio and heart-rate variability. For the measure of heart-rate variability you will be asked to wear a small ECG monitor and three small adhesive electrodes on your chest and abdomen whilst performing some light activities (such as moving from lying to sitting). You will then be asked to complete some questionnaires relating to your diet, alcohol intake, smoking and eating style.

Following the lab session you will be emailed a username and password which will enable you to log into our HealthValues website. You will be asked to log into the site on a weekly basis for a period of 24 weeks (approximately 6 months) to complete a series of tasks and/or questionnaires. Each of these weekly sessions should take approximately 5 to 45 minutes to complete. HALF of all people who take part will be allocated to an 'experimental' group. For these individuals the online sessions will include questionnaires that track healthy eating, along with new tasks designed to provide further support. The remaining HALF of participants will be allocated to a 'monitoring' group. For these individuals the online sessions will include the questionnaires that track healthy eating, but NOT the new tasks.

You will be invited back to the lab 3 months and 6 months later to repeat the physiological and questionnaire measures.

As a token of our appreciation, and to cover any travel expenses incurred, you will receive £10 upon completion of the first lab session, £25 upon completion of the second lab session and £50 upon completion of the third lab session. You will also accrue small amounts (to a total of £65) for each web session you complete. This money will be given to you at the third lab session.

What will the heart-rate measure tell you?
This measure will give us a very general indication of cardiac fitness. It is not a clinical assessment and thus will not identify any clinically significant heart rhythm problems.

What happens if I'm allocated to the 'monitoring' group?
You will be asked to attend the three lab sessions and log into the website on a weekly basis for 24 weeks. The website sessions will contain questionnaires to help monitor health. Monitoring health behaviours has been shown to be a useful tool for reaching health goals. At the end of the 24 weeks we will offer you the opportunity to also complete the additional tasks should you wish to do so.

What happens if I'm allocated to the 'experimental' group?
You will be asked to attend the three lab sessions and log into the website on a weekly basis for 24 weeks. The website sessions will give you access to the new health-related tasks that you will be asked to complete alongside the questionnaire measures.

What does the healthy eating programme consist of?
The programme consists of four main parts. In Part 1 we give you some feedback about your diet, along with some general dietary advice. In Part 2 we ask you to think a bit more deeply about health-related issues. In Part 3 we ask you to make specific plans to change particular behaviours. Finally, in Part 4 we give you options to repeat tasks, work on more plans and view weekly tips. All the sessions employed in the programme are based on psychological principles that have been shown to help individuals change their behaviour.

What kind of dietary feedback will you give me?
We will use some of the information you give us to provide you with estimates of your intake of fruit and vegetables, saturated fat and added sugar. However, it is important to be aware that these are only estimates, the accuracy of which may be influenced by a range of different factors. If you are concerned about your health, or the feedback you receive from the programme, you should contact your GP.

What type of health advice will you give me?
We will provide you with very general dietary advice. Again, if you have any concerns about your diet or your health it is important that you consult your GP. If you suffer from any conditions that effect your diet (e.g., pregnancy, diabetes, eating disorder), it is also important that you consult with your GP before making any changes.

Will the programme help me lose weight?
Whilst the programme has not been designed as a weight loss tool, the changes it advocates (i.e. eating more fruit and vegetables and less saturated fat and added sugar) are compatible with weight loss goals. As such we hope that individuals who are trying to lose weight will find it helpful.

What types of questionnaires will I complete online?
The questionnaires mainly relate to health behaviours such as diet and exercise. There are also some personality questionnaires. Additionally we ask you to provide some background information about yourself.

I only want to take part if I can complete the experimental healthy eating programme. Should I still sign up?
Unfortunately, in order to evaluate the programme properly, it is important that participants are **randomly** assigned to the 'experimental' or 'monitoring' group. For this reason, we cannot guarantee that you will be allocated to the experimental group. You will have a 50% chance of being allocated to this group. In other words, if 100 people sign up, we will ask 50 of them to complete the experimental programme. We appreciate that this makes your decision more difficult and hope that you won't be too disappointed should you not be asked to complete the programme. However, in order to evaluate the programme properly, it is also important that people don't change their minds about participating. For this reason we ask that you only sign up if you are prepared to be in either the 'experimental' or 'monitoring' group.

What happens if I sign up then change my mind?
It is important that you only sign up if you are willing and able to complete the three lab sessions and 24 weekly online sessions. However, should things change once you have signed up you are free to withdraw at any time. Simply contact a member of the HealthValues team on 07950 136663 or at healthvalues@swansea.ac.uk

What will happen to the information you collect?
All information collected will be confidential and will be kept securely in strict accordance with the Data Protection Act. It will not be used for any other purpose. An analysis of the information will form part of our report at the end of the study and may be presented to interested parties and published in scientific journals and related media.

What if there is something I don't understand or if I have further questions?
Please contact a member of the HealthValues team who will be very happy to discuss the project further with you. They can be reached on 07950 136663 or at healthvalues@swansea.ac.uk
