# Supplementary material for: Development and Preliminary Evaluation of an Internet-Based Healthy Eating Program: Randomized Controlled Trial
Source: J Med Internet Res. 2014 Oct 10;16(10):e231. doi: 10.2196/jmir.3534 (PMC4210956; doi:10.2196/jmir.3534)
Supplement: Supplementary file 3 [file jmir_v16i10e231_app3.pdf]

Appendix 3. Intervention components used in the HealthValues Programme together with most closely aligned categories of behaviour change technique according to the Behavior Change Technique Taxonomy. [66] Shaded rows indicate optional components.

Phase and session number	Intervention component	Behaviour change technique category
**Motivational**		
1	• Estimated intake of saturated fat, added sugar and fruit and vegetables	2.2
	• Information on recommended intake	1.6
	• Information on health consequences of a poor diet	5.1
	• Suggestions of ways to change diet	4.1
	• Links to additional information on government website	9.1
3	• Five minutes thinking about reasons for health values	Appropriate category not identified
5	• Five minutes thinking about health-related desires and aspirations and associated feelings	16.2
7	• Five minutes thinking about health-related concerns and associated feelings	5.5
8	• Estimated intake of saturated fat, added sugar and fruit and vegetables	2.2

	• Information on recommended intake	1.6
	• Opportunity to review dietary information and suggestions provided in Session 1	Replication of previous technique(s)
**Volitional**		
9	• Opportunity to review dietary information and suggestions provided in Session 1	Replication of previous technique(s)
	• Formation of one implementation intention using mental contrasting	1.1; 1.4; 7.1; 9.3; 15.2
	• Emailed reminders of implementation intention 1, 2 and 4 days after session completion	8.3
10	• User rating of implementation intention adherence followed by tailored response which may have included congratulations, encouragement, advise to seek social support, advise to use reminders	1.5; [10.4; 3.1; 7.1]
	• Opportunity to review previous implementation intention or create new one	8.3; 1.1; 1.4; 7.1; 9.3; 15.2
	• Opportunity to review dietary information and suggestions provided in Session 1	Replication of previous technique(s)
	• Emailed reminders of any new implementation intention 1, 2 and 4 days after session completion	8.3
11	• User rating of implementation intention adherence followed by tailored response which	1.5; [10.4; 3.1; 7.1]

	may have included congratulations, encouragement, advise to seek social support, advise to use reminders	
	• Opportunity to review previous implementation intention or create new one	8.3; 1.1; 1.4; 7.1; 9.3; 15.2
	• Opportunity to review dietary information and suggestions provided in Session 1	Replication of previous technique(s)
	• Emailed reminders of any new implementation intention 1, 2 and 4 days after session completion	8.3
**Maintenance**		
12	• Estimated intake of saturated fat, added sugar and fruit and vegetables	2.2
	• Information on recommended intake	1.6
	• Opportunity to review dietary information and suggestions provided in Session 1	Replication of previous technique(s)
	• Opportunity to review and revise tasks from Sessions 3, 5, and 7	Replication of previous technique(s)
	• Opportunity to review previous implementation intentions or create new ones (up to a total of 6).	8.3; 1.1; 1.4; 7.1; 9.3; 15.2
	• Emailed reminders of any new implementation intentions 1, 2 and 4 days after session completion	8.3
	• Opportunity to view the Tip of the Week	5.6

15	• Information on stimulus control	12.1; 12.3
13-23	• Opportunity to review estimated and recommended intake from Session 12	Replication of previous technique(s)
	• Opportunity to review dietary information and suggestions provided in Session 1	Replication of previous technique(s)
	• Opportunity to review and revise tasks from Sessions 3, 5, and 7	Replication of previous technique(s)
	• Opportunity to review previous implementation intentions or create new ones (up to a total of 6).	8.3; 1.1; 1.4; 7.1; 9.3; 15.2
	• Emailed reminders of any new implementation intentions 1, 2 and 4 days after session completion	8.3
	• Opportunity to view the Tip of the Week	12.1; 4.2; 8.2; 5.6; 12.6; appropriate category not identified; 3.1; 12.3; 12.5
	• Opportunity to review previous Tips	Replication of previous technique(s)
24	• Estimated intake of saturated fat, added sugar and fruit and vegetables	2.2
	• Information on recommended intake	1.6
	• Opportunity to review dietary information and suggestions provided in Session 1	Replication of previous technique(s)
	• Opportunity to review and revise tasks from Sessions 3, 5,	Replication of previous

and 7	technique(s)
• Opportunity to review previous implementation intentions or create new ones (up to a total of 6).	8.3; 1.1; 1.4; 7.1; 9.3; 15.2
• Emailed reminders of any new implementation intentions 1, 2 and 4 days after session completion	8.3
• Opportunity to view the Tip of the Week	Appropriate category not identified
• Opportunity to review previous Tips	Replication of previous technique(s)
