# Supplementary material for: Development and Preliminary Evaluation of an Internet-Based Healthy Eating Program: Randomized Controlled Trial
Source: J Med Internet Res. 2014 Oct 10;16(10):e231. doi: 10.2196/jmir.3534 (PMC4210956; doi:10.2196/jmir.3534)
Supplement: Supplementary file 4 [file jmir_v16i10e231_app4.pdf]

CONSORT-EHEALTH Checklist V1.6.2 Report

(based on CONSORT-EHEALTH V1.6), available at [http://tinyurl.com/consort-ehealth-v1-6].

3534

Date completed

5/14/2014 7:42:36

by

Tapper

Development and preliminary evaluation of an internet-based healthy eating programme: A randomised controlled trial.

TITLE

1a-i) Identify the mode of delivery in the title

Development and preliminary evaluation of an internet-based healthy eating programme: A randomised controlled trial.'

1a-ii) Non-web-based components or important co-interventions in title

n/a (Study does not include non-web-based components.)

1a-iii) Primary condition or target group in the title

n/a (Study does not have a specific target group.)

ABSTRACT

1b-i) Key features/functionalities/components of the intervention and comparator in the METHODS section of the ABSTRACT

The HealthValues Healthy Eating Programme is an internet-based intervention that employs a novel strategy for promoting behaviour change (analysing one's reasons for endorsing health values) alongside other psychological principles that have been shown to influence behaviour. The programme consists of phases targeting motivation (dietary feedback and advice, analysing reasons for health values, thinking about health-related desires and concerns), volition (implementation intentions with mental contrasting) and maintenance (reviewing tasks, weekly 'tips').'
'The programme was designed such that participants logged onto a website every week for 24 weeks and completed health-related measures. Those allocated to the intervention group also completed the intervention tasks at these sessions. Additionally, all participants attended laboratory sessions at baseline, 3 months and 6 months. '

1b-ii) Level of human involvement in the METHODS section of the ABSTRACT

The programme was designed such that participants logged onto a website every week for 24 weeks and completed health-related measures. Those allocated to the intervention group also completed the intervention tasks at these sessions. Additionally, all participants attended laboratory sessions at baseline, 3 months and 6 months. During these sessions, participants completed a food frequency questionnaire (FFQ, the Block Fat/Sugar/Fruit/Vegetable Screener, adapted for the UK), and researchers (blind to group allocation) measured their body mass index (BMI), waist-to-hip ratio (WHR) and heart rate variability (HRV).'

1b-iii) Open vs. closed, web-based (self-assessment) vs. face-to-face assessments in the METHODS section of the ABSTRACT

A total of 82 females and 18 males were recruited using both online and print advertisements in the local community.'
'The programme was designed such that participants logged onto a website every week for 24 weeks and completed health-related measures. Those allocated to the intervention group also completed the intervention tasks at these sessions. Additionally, all participants attended laboratory sessions at baseline, 3 months and 6 months. During these sessions, participants completed a food frequency questionnaire (FFQ, the Block Fat/Sugar/Fruit/Vegetable Screener, adapted for the UK), and researchers (blind to group allocation) measured their body mass index (BMI), waist-to-hip ratio (WHR) and heart rate variability (HRV).'

1b-iv) RESULTS section in abstract must contain use data

A total of 82 females and 18 males were recruited using both online and print advertisements in the local community.'
'Per protocol analysis (n = 92) showed...'

1b-v) CONCLUSIONS/DISCUSSION in abstract for negative trials

n/a. (Per-protocol analysis showed a significant effect on one of the (3) primary outcomes.

INTRODUCTION

2a-i) Problem and the type of system/solution

A diet that is high in saturated fat and added sugars and low in fruit and vegetables is associated with a range of chronic diseases....'
'One way of promoting a more healthy diet is via internet-based intervention. This has a range of potential advantages....'
'This paper describes the initial evaluation of a new, fully automated internet-based healthy eating intervention: the 'HealthValues Healthy Eating Programme'. This programme differs from previous web interventions in its use of novel behaviour change techniques...'

2a-ii) Scientific background, rationale: What is known about the (type of) system

...a number of fully automated internet-interventions have shown positive effects on diet...'

METHODS

3a) CONSORT: Description of trial design (such as parallel, factorial) including allocation ratio

The main aim of the study was to examine the effects of the programme on different types of health-related eating behaviours; those that require engagement (eating more fruit and vegetables) and those that require disengagement (eating less saturated fat and added sugar). However, we were also interested in examining 'spill-over' effects to other health-related behaviours (physical activity, alcohol consumption, smoking).'

3b) CONSORT: Important changes to methods after trial commencement (such as eligibility criteria), with reasons

n/a (There were no changes to methods after trial commencement.)

3b-i) Bug fixes, Downtimes, Content Changes

n/a (There were no bug fixes, downtimes or content changes after trial commencement.)

4a) CONSORT: Eligibility criteria for participants

As inclusion criteria, we stipulated that participants were aged 18 or over and able to comply with the study procedures (i.e., attend the laboratory appointments and complete the weekly online sessions). Other exclusion criteria were pregnancy, being out of the country for more than 3 weeks during the study period, another household member already participating, and participation in a previous related study.'

4a-i) Computer / Internet literacy

As inclusion criteria, we stipulated that participants were aged 18 or over and able to comply with the study procedures (i.e., attend the laboratory appointments and complete the weekly online sessions).'

4a-ii) Open vs. closed, web-based vs. face-to-face assessments:

Participants were recruited using both online and print advertisements in the local community. These included posters and flyers in local shops and community facilities, and advertisements on social media sites, email networks and in local newspapers. The advertisements stated that the study team were looking for individuals to test a new online healthy eating programme and noted that individuals would be reimbursed for participation. The study's website address (which included a full participant information sheet) was included in the advertisement.'
Details of whether measures were assessed online, or by researchers in the laboratory, are given throughout the 'Outcome Measures' section.

4a-iii) Information giving during recruitment

Example advertisement and information sheet are provided as an appendix.

4b) CONSORT: Settings and locations where the data were collected

Details of whether each measure was assessed in the laboratory, or online, are provided through the 'Outcome Measures' section.

4b-i) Report if outcomes were (self-)assessed through online questionnaires

Details of whether each measure was self- or researcher-assessed are provided through the 'Outcome Measures' section.

4b-ii) Report how institutional affiliations are displayed

We have no particular reason to believe this would bias our results. As is standard, University affiliations were indicated in recruitment and information material, but not highlighted.

5) CONSORT: Describe the interventions for each group with sufficient details to allow replication, including how and when they were actually administered

5-i) Mention names, credential, affiliations of the developers, sponsors, and owners

The intervention was developed by the authors. This is indicated throughout the paper by the use of personal pronouns.

5-ii) Describe the history/development process

Development and preliminary evaluation of an internet-based healthy eating programme...'
'The intervention was tested for usability prior to the study.'

5-iii) Revisions and updating

n/a (Intervention was not revised or updated during the study. There were no dynamic components.)

5-iv) Quality assurance methods

n/a (See above.)

5-v) Ensure replicability by publishing the source code, and/or providing screenshots/screen-capture video, and/or providing flowcharts of the algorithms used

The study was designed as a preliminary evaluation, with a view to inform further programme development. As such, we do not feel it would be worthwhile conducting additional evaluations of the complete programme in its present form. Researchers may wish to replicate individual programme components and in this instance may contact the first author for screenshots. (Screenshots of the entire programme would be numerous and are likely to be unnecessary.)

5-vi) Digital preservation

Programme homepage is www.healthvalues.co.uk
A screenshot of the homepage as it appeared during the study in contained in the Appendix.

Please also see answer to question 5-v.

5-vii) Access

Each participant received £10 for attending the first laboratory session, £25 for the second and £50 for the third. Additionally they received £2 per session for completing the first ten online sessions, £2.50 per session for completing the next ten online sessions and £5 per session for completing the last four online sessions. Thus, participants could receive up to £150 for completing all laboratory and online sessions.'

We did not ask participants how they accessed the intervention.

Interested individuals are welcome to contact the first author to obtain access to a demonstration of the intervention. (Though we are not able to guarantee availability.)

5-viii) Mode of delivery, features/functionalities/components of the intervention and comparator, and the theoretical framework

Please see Appendix 1.

5-ix) Describe use parameters

All participants were asked, by automated email, to log onto the study website every week on 24 separate occasions to complete measures (intervention and control group) and programme tasks (intervention group only). Each session could be accessed 6 days after completion of the previous session. Once the session became available the participant was sent an email asking them to log in to complete it. Up to three automated reminders were emailed two, four and six days later to participants who had failed to complete the session. After completion of each session the participant was sent an automated email thanking them and reminding them to log in again the following week.'

5-x) Clarify the level of human involvement

Whilst researchers were involved in the evaluation of the intervention, the intervention itself is designed to be stand-alone.

'The HealthValues Healthy Eating Programme is a stand-alone, internet-based intervention...'

5-xi) Report any prompts/reminders used

Once the session became available the participant was sent an email asking them to log in to complete it. Up to three automated reminders were emailed two, four and six days later to participants who had failed to complete the session. After completion of each session the participant was sent an automated email thanking them and reminding them to log in again the following week. Where participants failed to login for 3 weeks GJB attempted to contact them by phone and then email to establish whether they still wanted to participate in the online sessions and, if not, to assure them that we would still be keen for them to attend the laboratory assessments.'

5-xii) Describe any co-interventions (incl. training/support)

n/a (The intervention was designed as stand-alone.)

6a) CONSORT: Completely defined pre-specified primary and secondary outcome measures, including how and when they were assessed

These details are provided in the 'Outcome measures' section.

6a-i) Online questionnaires: describe if they were validated for online use and apply CHERRIES items to describe how the questionnaires were designed/deployed

All online questionnaires were tested for usability prior to the study. Questionnaires and items were presented in the same order for each participant and participants needed to complete all items before progressing to the next screen. Adaptive questioning was used for the IPAQ.'

6a-ii) Describe whether and how “use” (including intensity of use/dosage) was defined/measured/monitored

Please see Figure 1 for use statistics.

6a-iii) Describe whether, how, and when qualitative feedback from participants was obtained

Data relating to potential mediators, moderators and process measures were also collected but these are not discussed in the present paper.'

6b) CONSORT: Any changes to trial outcomes after the trial commenced, with reasons

There were no changes to trial outcomes after the trial commenced.

7a) CONSORT: How sample size was determined

7a-i) Describe whether and how expected attrition was taken into account when calculating the sample size

Given that this study serves as an initial test of the programme, there were no comparable studies on which to base sample size calculations. That said, our sample size was informed by our previous research that examined the effects of one of the intervention components (thinking about reasons for values) on eating behaviour over a 7-day period.[24] The eating behaviour measure showed a mean difference between groups of 0.92 and a standard deviation of 1.51, meaning that at 80% power, 44 participants per group would be needed to detect a significant difference (two-tailed, $P < .05$). Assuming an attrition rate of no more than 15%,[47] we concluded that a sample size of 100 would be appropriate for this exploratory trial.'

7b) CONSORT: When applicable, explanation of any interim analyses and stopping guidelines

There were no interim analyses.

8a) CONSORT: Method used to generate the random allocation sequence

KT then allocated participants to an intervention or control ('monitoring') group using a stratified block randomisation protocol on the basis of dieting status (dieting versus non-dieting) and fruit and vegetable consumption (5 or more portions a day versus less than 5 a day). Block size was 2 and random numbers were generated in Excel.'

8b) CONSORT: Type of randomisation; details of any restriction (such as blocking and block size)

KT then allocated participants to an intervention or control ('monitoring') group using a stratified block randomisation protocol on the basis of dieting status (dieting versus non-dieting) and fruit and vegetable consumption (5 or more portions a day versus less than 5 a day). Block size was 2 and random numbers were generated in Excel.'

9) CONSORT: Mechanism used to implement the random allocation sequence (such as sequentially numbered containers), describing any steps taken to conceal the sequence until interventions were assigned

KT then emailed the participant details of their user ID and password and they were informed of their group allocation the first time they logged on.'

10) CONSORT: Who generated the random allocation sequence, who enrolled participants, and who assigned participants to interventions

Following baseline assessment GJB emailed KT details of each participant's dieting status and fruit and vegetable consumption. KT then allocated participants to an intervention or control ('monitoring') group using a stratified block randomisation protocol on the basis of dieting status (dieting versus non-dieting) and fruit and vegetable consumption (5 or more portions a day versus less than 5 a day). Block size was 2 and random numbers were generated in Excel. KT then emailed the participant details of their user ID and password and they were informed of their group allocation the first time they logged on.'

11a) CONSORT: Blinding - If done, who was blinded after assignment to interventions (for example, participants, care providers, those assessing outcomes) and how

11a-i) Specify who was blinded, and who wasn't

Laboratory measures were taken at baseline (February to April), 3 months (May to July) and 6 months (August to October) by GJB and a second research assistant, both of whom were blind to group allocation.'

'Although participants were not blind to group allocation they were informed that both the 'experimental' group and the 'monitoring' group would monitor eating behaviours and that this had been shown to be useful for reaching health goals.'

11a-ii) Discuss e.g., whether participants knew which intervention was the "intervention of interest" and which one was the "comparator"

Although participants were not blind to group allocation they were informed that both the 'experimental' group and the 'monitoring' group would monitor eating behaviours and that this had been shown to be useful for reaching health goals.'

11b) CONSORT: If relevant, description of the similarity of interventions

All participants were asked, by automated email, to log onto the study website every week on 24 separate occasions to complete measures (intervention and control group) and programme tasks (intervention group only).'

12a) CONSORT: Statistical methods used to compare groups for primary and secondary outcomes

A series of 3 x 2 mixed ANOVA models were used to examine the effects of the intervention on lab measured intake of (a) saturated fat, (b) added sugar and (c) fruit and vegetables. Independent variables were time (baseline, 3 months, 6 months) and group (control, intervention).'

'Per protocol analysis was conducted on all primary and secondary outcomes by including only those participants who completed all three laboratory assessments as well as 12 or more of the 24 online sessions (for laboratory measures) or all 24 online sessions (for online measures). A series of 3 (time) x 2 (group) mixed ANOVA models were used to examine effects on lab-based measures whilst 4 (time) x 2 (group) ANOVA models were used for online measures.'

'Fishers exact test was used to examine smoking status and Chi-square was used for binge drinking status.'

12a-i) Imputation techniques to deal with attrition / missing values

Missing data were replaced by calculating the mean change from previous observations in the control group and adding or subtracting this figure from the previous observation relating to the missing data point.'

12b) CONSORT: Methods for additional analyses, such as subgroup analyses and adjusted analyses

Per protocol analysis was conducted on all primary and secondary outcomes by including only those participants who completed all three laboratory assessments as well as 12 or more of the 24 online sessions (for laboratory measures) or all 24 online sessions (for online measures). A series of 3 (time) x 2 (group) mixed ANOVA models were used to examine effects on lab-based measures whilst 4 (time) x 2 (group) ANOVA models were used for online measures. Analyses were conducted with outliers (defined as 3.5 SDs from the mean) both included and excluded. Fishers exact test was used to examine smoking status and Chi-square was used for binge drinking status.'

'To examine the effects of the individual intervention strategies employed in the motivational phase, change scores were calculated using the dietary behaviours questionnaire. These were computed using figures from the session in which the strategy was employed and two sessions later (e.g., change between Sessions 1 and 3, see Appendix 1 for details of strategies). Change score was then employed as the dependent variable in a 2 (condition) x 4(strategy) mixed ANOVA.'

RESULTS

13a) CONSORT: For each group, the numbers of participants who were randomly assigned, received intended treatment, and were analysed for the primary outcome

See Figure 1, Table 2 and Table 3.

13b) CONSORT: For each group, losses and exclusions after randomisation, together with reasons

See Figure 1.

13b-i) Attrition diagram

See Figure 1.

14a) CONSORT: Dates defining the periods of recruitment and follow-up

Laboratory measures were taken at baseline (February to April), 3 months (May to July) and 6 months (August to October)...'

14a-i) Indicate if critical "secular events" fell into the study period

n/a (No such events fell into the study period.)

14b) CONSORT: Why the trial ended or was stopped (early)

n/a (The trial was not stopped early.)

15) CONSORT: A table showing baseline demographic and clinical characteristics for each group

Please see Table 1.

15-i) Report demographics associated with digital divide issues

Please see Table 1.
16a) CONSORT: For each group, number of participants (denominator) included in each analysis and whether the analysis was by original assigned groups
16-i) Report multiple “denominators” and provide definitions
Per protocol analysis was conducted on all primary and secondary outcomes by including only those participants who completed all three laboratory assessments as well as 12 or more of the 24 online sessions (for laboratory measures) or all 24 online sessions (for online measures).'
See also Table 3.
16-ii) Primary analysis should be intent-to-treat
Given the exploratory nature of the trial, intention-to-treat analyses were conducted on primary outcomes only.'
'Per protocol analysis was conducted on all primary and secondary outcomes by including only those participants who completed all three laboratory assessments as well as 12 or more of the 24 online sessions (for laboratory measures) or all 24 online sessions (for online measures).'
17a) CONSORT: For each primary and secondary outcome, results for each group, and the estimated effect size and its precision (such as 95% confidence interval)
See Tables 2 and 3.
17a-i) Presentation of process outcomes such as metrics of use and intensity of use
We have excluded the discussion of process data from this paper. ('Data relating to potential mediators, moderators and process measures were also collected but these are not discussed in the present paper.')
17b) CONSORT: For binary outcomes, presentation of both absolute and relative effect sizes is recommended
Analysis of binge drinking included 90 participants who provided data on alcohol consumption at all three laboratory assessments and completed at least 12 of the online sessions. Again, at each of the three time-points, there was no difference in the proportion of individuals who engaged in binge drinking in the experimental group compared to the control group. (Baseline: control n = 25, experimental n = 23, 2 = 0.04, P = .84; 3 months: control n = 23, experimental n = 17, 2 = 1.18, P = .28; 6 months: control n = 20, experimental n = 17, 2 = 0.22, P = .64.)'
18) CONSORT: Results of any other analyses performed, including subgroup analyses and adjusted analyses, distinguishing pre-specified from exploratory
Because fruit and vegetable consumption was improved by the intervention, we conducted exploratory analyses examining changes in fruit and vegetable consumption in the intervention and control groups in the two-week period following the delivery of each of the four different programme components (see Figure 3). There was no main effect of strategy, F(1, 91) = 0.53, P = .47, = 0.01 or condition, F(1, 91) = 0.87, P = .47, = 0.01 and no significant interaction between strategy and condition, F(1, 91) = 2.88, P = .09, = 0.03 (though the latter results are marginal).'
18-i) Subgroup analysis of comparing only users
Although the samples for such analyses are subject to bias, they are an important means of examining intervention efficacy in exploratory trials.'
19) CONSORT: All important harms or unintended effects in each group
n/a (There were no harms or unintended effects.)
19-i) Include privacy breaches, technical problems
There were no privacy breaches.
Two participants (both control group) experienced difficulties logging in part-way through the programme. Both were advised to try logging in using a different computer. One of these participants experienced no further problems and went on to complete all sessions. The other participant continued to experience problems and only completed up to Session 7.
A third participant (experimental group) experienced difficulties whilst using the programme. This participant was assigned a new login and password and went on to complete 7 sessions.
19-ii) Include qualitative feedback from participants or observations from staff/researchers
We have not included these data since we felt it would make the manuscript too lengthy.

DISCUSSION

20) CONSORT: Trial limitations, addressing sources of potential bias, imprecision, multiplicity of analyses
20-i) Typical limitations in ehealth trials
In future research it would be important to trial the programme in the absence of incentives for session completion...'
21) CONSORT: Generalisability (external validity, applicability) of the trial findings
21-i) Generalizability to other populations
It would also be important to examine the effects of the programme with different populations....'
21-ii) Discuss if there were elements in the RCT that would be different in a routine application setting
In future research it would be important to trial the programme in the absence of incentives for session completion. Given the high rates of attrition in online interventions[79] we incorporated these incentives to enable a proper initial evaluation of the programme. However, a trial without these incentives would help indicate natural attrition and allow for calculations of cost-effectiveness.'
22) CONSORT: Interpretation consistent with results, balancing benefits and harms, and considering other relevant evidence
22-i) Restate study questions and summarize the answers suggested by the data, starting with primary outcomes and process outcomes (use)
Please see Discussion section.
22-ii) Highlight unanswered new questions, suggest future research
...Comparing different versions of the programme would help distinguish between these possibilities.'
'In future research it would be important to trial the programme in the absence of incentives for session completion....'
'It would also be important to examine the effects of the programme with different populations...'

Other information

23) CONSORT: Registration number and name of trial registry
Although the study was a randomised controlled trial design, given its exploratory nature the trial was not registered.'
24) CONSORT: Where the full trial protocol can be accessed, if available
Please see answer to question 23. Further details may be obtained by contacting the first author.
25) CONSORT: Sources of funding and other support (such as supply of drugs), role of funders
The research was funded by the Economic and Social Research Council.'
X26-i) Comment on ethics committee approval
The study received ethics approval from Swansea University Psychology Department Ethics Committee.'
x26-ii) Outline informed consent procedures
Informed consent was collected by researchers at the first laboratory assessment (see below).'
X26-iii) Safety and security procedures
Participants were asked to contact their GP should they have any concerns about their health or diet. This advice was provided in the information sheet at the start of the study and also online, at relevant points of the intervention.
X27-i) State the relation of the study team towards the system being evaluated

KT, GM, GH and GJB were responsible for the development of the programme. (This is indicated in the manuscript by use of personal pronouns.)
